# Draft Genome Sequence of *Streptomyces* sp. JHA26, a Strain That Harbors a PA14 Domain Containing β-d-Galactofuranosidase

**DOI:** 10.1128/genomeA.00190-17

**Published:** 2017-04-13

**Authors:** Emiko Matsunaga, Yujiro Higuchi, Kazuki Mori, Kosuke Tashiro, Kaoru Takegawa

**Affiliations:** Department of Bioscience and Biotechnology, Faculty of Agriculture, Kyushu University, Hakozaki, Fukuoka, Japan

## Abstract

The genome sequence of *Streptomyces* sp. strain JHA26, the culture supernatant of which exhibited β-d-galactofuranosidase (Gal*f*-ase) activity, was analyzed to search for a Gal*f*-ase-encoding gene. We report here the results of whole-genome shotgun sequencing and reveal the identity of a new Gal*f*-ase gene.

## GENOME ANNOUNCEMENT

In some bacterial and fungal species, β-d-galactofuranose (Gal*f*) is found as a constituent of polysaccharides and glycoconjugates (1–5). Although there are several studies reporting on the molecular mechanism of Gal*f* biosynthesis, there are only a few reports on Gal*f* metabolism (6). We previously identified a gene, termed ORF1110, encoding a Gal*f*-specific β-d-galactofuranosidase (Gal*f*-ase) (7, 8). This Gal*f*-ase gene was found in the genome of *Streptomyces* sp. strain JHA19, which was isolated by screening for soil microbes that exhibited Gal*f*-ase activity using *4*-nitrophenyl β-d-galactofuranoside (*p*NP-β-d-Gal*f*) as the substrate. We found another *Streptomyces* sp. strain, named JHA26, which also exhibited Gal*f*-ase activity. Here, we report the draft genome sequence of strain JHA26.

The draft sequence was obtained by a whole-genome shotgun sequencing strategy. Sequencing was carried out using Ion PGM (Life Technologies), GS FLX Titanium (Roche), and GS FLX (Roche) sequencers. With an average single-read sequencing length of 193 bp, 316 Mb of sequence data were generated from 1.39 × 10^6^ sequencing reads (43.1-fold coverage). These sequence reads were assembled using Newbler version 2.7 software, and 170 contigs (>500 bp) were generated, the longest of which contained 291,049 bp. To search for genes with predicted functions, genome annotation was carried out using Glimmer version 3.02b software and the nonredundant protein sequence database of BLAST version 2.2.26. InterProScan version 4.8 software was also used for finding glycosyl hydrolases (GHs). The total draft genome size of *Streptomyces* sp. JHA26 was 7.34 Mb, with an average G+C content of 72.5% and containing 6,748 genes. The mean and median gene lengths were 929 bp and 801 bp, respectively, and the gene density was 1,088 bp/gene.

The genome annotation and homology search revealed that strain JHA26 harbored a predicted Gal*f*-ase gene, designated here as ORF0643, located in contig003 (GenBank accession no. BDJC01000003); this gene was homologous to the ORF1110 Gal*f*-ase gene of strain JHA19. By using the Pfam program (http://pfam.xfam.org), it was predicted that the ORF0643 Gal*f*-ase contained GH2-related domains, as was also found for the ORF1110 Gal*f*-ase. Apart from the GH2-related domains, the ORF0643 Gal*f*-ase also contained a protective antigen 14 (PA14) domain in the N-terminus; the ORF1110 Gal*f*-ase, on the other hand, contained an α-l-arabinofuranosidase domain B (AbfB) in the C-terminus. Each of those discrete domains was thought to be involved in substrate recognition. However, we found that the AbfB domain of ORF1110 was dispensable for the Gal*f*-ase activity of both substrates of artificial *p*NP-β-d-Gal*f* and natural *Aspergillus fumigatus* galactomannan (8). In contrast, we found that the PA14 domain of ORF0643 was important for the Gal*f*-ase activity of *A. fumigatus* galactomannan (9). We have also found that there are two types of Gal*f*-ases among the *Actinobacteria*: one type containing the AbfB domain and the other type containing the PA14 domain (9).

### Accession number(s).

The contig sequences of *Streptomyces* sp. strain JHA26 have been deposited at DDBJ/EMBL/GenBank under the accession numbers BDJC01000001 to BDJC01000170.
